# The roadmap towards elimination of lymphatic filariasis by 2030: insights from quantitative and mathematical modelling

**DOI:** 10.12688/gatesopenres.13065.1

**Published:** 2019-09-13

**Authors:** NTD Modelling Consortium Lymphatic Filariasis Group

**Keywords:** Lymphatic filariasis, Elimination, NTD Modelling Consortium, mass drug administration, modelling, Sustainable Development Goals, feasibility

## Abstract

The Global Programme to Eliminate Lymphatic Filariasis was launched in 2000 to eliminate lymphatic filariasis (LF) as a public health problem by 1) interrupting transmission through mass drug administration (MDA) and 2) offering basic care to those suffering from lymphoedema or hydrocele due to the infection. Although impressive progress has been made, the initial target year of 2020 will not be met everywhere. The World Health Organization recently proposed 2030 as the new target year for elimination of lymphatic filariasis (LF) as a public health problem. In this letter, LF modelers of the Neglected Tropical Diseases (NTDs) Modelling Consortium reflect on the proposed targets for 2030 from a quantitative perspective. While elimination as a public health problem seems technically and operationally feasible, it is uncertain whether this will eventually also lead to complete elimination of transmission. The risk of resurgence needs to be mitigated by strong surveillance after stopping interventions and sometimes perhaps additional interventions.

## Disclaimer

The views expressed in this article are those of the authors and do not necessarily reflect the views of the World Health Organization. Publication in Gates Open Research does not imply endorsement by the Gates Foundation.

## Background

Lymphatic filariasis (LF) is a mosquito-borne neglected tropical disease (NTD) that is caused by the filarial parasites *Wuchereria bancrofti*, *Brugia malayi* and *B. timori* and occurs worldwide in tropical and subtropical areas. Infection can lead to lymphoedema, elephantiasis and hydrocele; all severely disabling, chronic conditions. Recognizing the huge socio-economic burden caused by LF and considering advances in diagnosis and treatment, the World Health Assembly adopted Resolution 50.29 in 1997, calling for global elimination of LF as a public health problem ^1^. To achieve this goal, the Global Programme to Eliminate Lymphatic Filariasis (GPELF) was launched in 2000, with the following specific targets for 2020: 1) to interrupt transmission by annual mass drug administration (MDA) with two-drug combinations of donated antifilarial drugs, and 2) to alleviate the suffering of those affected with lymphoedema and hydrocele by offering a basic package of care ^2^. The fight against LF and other NTDs was further reinforced by the London Declaration on Neglected Tropical Diseases in 2012 ^3^ and by the adoption of the United Nations sustainable development goals (SDGs) for 2030, which include the goal to end the epidemic of neglected tropical diseases ^4^.

Important progress towards the goals has been made, with eleven countries having validated elimination of LF as a public health problem by 2017 ^2^, meaning that criteria for both GPELF targets were met. In addition, ten countries were under post-treatment surveillance after having reached criteria for stopping MDA in all endemic districts, and 32 had scaled-up MDA to all districts in need of treatment. However, there were also five countries that had not yet started MDA in any of the endemic districts and thirteen countries that are treating only part of the districts in need of MDA. Moreover, in many countries, the recommended basic package of care for people with lymphoedema or hydrocele is not yet universally available. Clearly, GPELF’s 2020 targets will not be met everywhere.

In consultation with the global NTD community, the World Health Organization (WHO) is currently developing new targets and milestones beyond 2020, which should be aligned with the sustainable development goals (SDGs) and should be ambitious, evidence-based and realistic ^5^. Endemic country representatives, implementing partners, donors and other stakeholders were invited to provide feedback on WHO proposed milestones and targets during two rounds of online consultations (April–July 2019). For LF, WHO proposes to keep the global elimination of LF as a public health problem as the main goal, with an adapted timeline. By 2030, all countries should have completed their MDA programs, should be implementing post-MDA or post-validation surveillance, and should have implemented a minimum package of care for LF morbidity ^6^.

Members of the NTD Modelling Consortium were also included in the consultation process. The NTD Modelling Consortium was set up in 2014 with funding from the Bill & Melinda Gates Foundation to support ongoing efforts to control and eliminate NTDs by high-quality quantitative modelling ^7^. Within this consortium, modelers working on various NTDs joined forces to address the most pressing policy questions and to accelerate innovations in the mathematical modelling of NTDs by exchanging ideas and insight. Among the consortium’s key outputs is a detailed assessment across NTDs, including LF, of whether WHO’s 2020 goals can be met with current strategies and where acceleration strategies are required ^8, 9^.

In this Open Letter, we - LF specialists associated with the NTD Modelling Consortium - reflect on the proposed targets for 2030, drawing from our collective experience and modelling work by ourselves and others: how can the proposed targets be measured, are they technically and operationally feasible, what is needed to sustain the achievements, what are the main uncertainties, and what are the main risks to be mitigated in order to achieve and maintain the stated goals? A summary of key points is provided in Table 1.

**Table 1.** Modelling insights and the feasibility of the proposed WHO 2030 targets for LF and the main challenges.

Current WHO Goal	Elimination as a public health problem (<1% microfilaria prevalence) by 2020.
2030 Target	Global elimination as a public health problem by 2030.
Is the new target technically feasible under the current disease strategy?	Yes, provided that coverage is high enough, systematic non-adherence is low, and mass drug administration (MDA) has already started.
If not, what is required to achieve the target? (updated strategy, use of new tools, etc.)	For late-starting programs: optimize coverage, use annual MDA with triple drug or biannual MDA.
Are current tools able to reliably measure the target?	Yes. Elimination as a public health problem is defined as achieved when countries have passed three consecutive transmission assessment surveys (TAS 1-3) with a pre-defined methodology.
What are the biggest unknowns?	Where and when will passing TAS lead to elimination of transmission? Are additional interventions (e.g. vector control) required to main the achievements?
What are the biggest risks?	Risk of resurgence after passing TAS and stopping MDA.

## Models for lymphatic filariasis

Mathematical models for infectious disease provide a mechanistic, quantitative representation of the processes involved in transmission and control, and they can be used to predict the impact of interventions or to forecast future events. Several LF models are applied within the NTD modelling consortium, named EPIFIL, LYMFASIM and TRANSFIL, each with their own strengths and limitations. All models dynamically simulate LF transmission and the impact of interventions in a closed population, usually representing a village or town. However, the models are different in details, employed modelling technique, and how they are mostly applied. EPIFIL is a deterministic, population-based model, which is nowadays implemented within a Monte Carlo-based Bayesian melding framework to fit the model to local data, while capturing the remaining uncertainties in estimated parameters ^10, 11^. LYMFASIM ^12, 13^ and TRANSFIL ^14^ are both individual-based, stochastic models, meaning that individuals in the population are explicitly represented with their own characteristics to capture within-population heterogeneities. These models can be computationally intensive, making calibration a time-consuming process, and usually the value of most parameters is fixed in model applications. Models and modelling methods are continuously improved and refined to deal with new research questions. Recent advances include the development of model-ensembling approaches (to combine predictions from multiple models) ^15^ and efforts to capture geospatial heterogeneities ^10, 16^. Outside of the modelling consortium, a geospatially-explicit model was recently developed for American Samoa, that captures the connectedness between sites via migrating humans ^17^. For an explanation of modelling terminology, we refer to a recently published glossary ^18^.

## Insights gained from modelling analyses

### Measuring the target

WHO considers LF to be eliminated as a public health problem, if periodic transmission assessment surveys (TAS, with a predefined survey design) show that the average infection prevalence has been reduced and sustained below a critical threshold, expecting that transmission will eventually cease and the risk of resurgence is minimal ^19^. In areas with bancroftian filariasis transmitted by *Anopheles* or *Culex*, the critical threshold has been set at 1% microfilaria (mf) prevalence in the community or 2% antigen prevalence in 6–7 year-old children; slightly lower values are used where *Aedes* is the main vector of bancroftian filariasis; for brugian filariasis, 2% antibody prevalence is used as critical threshold. Passing TAS does not necessarily mean that infection prevalence is below the threshold across the entire district; small foci with low-level residual transmission can be missed by TAS-like surveys, and additional effort is needed to detect microfoci ^20^. Uncertainty about the dynamics of, and association between, different infection indicators ^21^ makes it difficult to quantify the risk of resurgence associated with signals of residual transmission.

### Timeline to achieve the target and technical feasibility

Models have been used to examine timelines to achieving elimination as a public health problem, usually defined as mf prevalence below 1%. Modelling suggests that achieving the 1% mf prevalence target is technically feasible with the standard WHO-recommended strategy of annual MDA with a two-drug combination (diethylcarbamazine + albendazole (DA) or ivermectin +albendazole (IA)). However, the required treatment duration strongly depends on baseline endemicity and achieved coverage (here defined as percentage treated out of the total population) and may often exceed the initially anticipated 5–6 years ^9^. Poor coverage severely impedes elimination programs, especially when a large group of people is systematically not treated in repeated MDA rounds (also called systematic non-adherence or systematic non-compliance) ^9, 22^. The risk of not achieving the 2030 targets is highest in areas with late-start MDA, high local baseline prevalence, and/or insufficient coverage.

Models were also used to explore to what extent elimination can be accelerated by using alternative strategies. Firstly, the required treatment duration can be minimized by optimizing the coverage and preventing systematic non-adherence ^9, 23, 24^. This will enhance the impact per round and reduce the risk that residual transmission persists in an untreated population subgroup. This is particularly important for areas with a history of poor coverage. Secondly, treatment duration can likely be reduced by treating with more efficacious treatment regimens, such as a triple-drug combination of ivermectin, diethylcarbamazine and albendazole (IDA). This triple-drug combination was shown to be more efficacious than the standard two-drug regimens ^25^ and our modelling suggested that the required treatment duration can be reduced by a third by using IDA instead of DA ^9, 26^. However, using IDA is not a solution for poor coverage. Additionally, IDA is not safe in areas endemic for onchocerciasis or loiasis, and therefore cannot be used in large parts of Africa. The use of DEC-medicated salt can also be highly efficacious, but will require a completely different treatment delivery approach ^27^. Thirdly, models predicted that the required treatment duration can also be reduced considerably by treating biannually (i.e. twice per year) instead of annually if coverage remains the same, assuming that a) the second round reaches some people who were missed in the first round and b) people treated twice benefit from additional chemotherapeutic effects on worms and mf ^9, 23, 24^. However, these predictions were not confirmed by recent community intervention trials and concerns exists on feasibility of biannual campaigns in low-resources settings. Lastly, models showed that complementary vector control (enhanced coverage of insecticide-treated bednets) has little impact on the required programme duration for reducing mf prevalence below 1%, but will help to reduce risk of resurgence ^24, 26^. In 2017, WHO issued new guidelines on the use of alternative MDA regimens for LF elimination, informed by empirical data and modelling ^28^. They recommend the use of IDA in onchocerciasis and loiasis-free areas that have not started MDA or have not yet had four rounds with effective coverage (i.e. >65% of the total population), and for areas that failed to meet epidemiological thresholds for elimination as a public health after five or more treatment rounds with effective coverage. The use of biannual MDA is not recommended.

Detailed predictions of when elimination of LF as a public health problem can be achieved in African countries, under current or alternative strategies, have been published elsewhere ^24^. Accurate prediction is often difficult due to geospatial variation in and uncertainty about baseline endemicity and achieved coverage levels. Programmatic data on coverage are often unreliable due to different factors (e.g. not everyone who receives a tablet may also swallow, uncertainty about the overall population size makes it difficult to estimate coverage as percentage, health workers and/or programme managers at different levels may be incentivized to report inflated coverage figures). Data from sentinel sites can be used to validate and constrain models ^29^.

### Operational feasibility

A key challenge will be ensuring high effective coverage. This can be done, as shown in various studies ^30– 32^, but preventing systematic non-adherence remains important. Although models suggest that treating biannually can be very effective to accelerate elimination, there can be a reluctance to adopt biannual MDA due to costs and logistics, so it may not always be feasible to implement. Moreover, biannual MDA at a lower coverage could exaggerate the effects of systematic non-adherence, whereas increasing coverage will decrease heterogeneity ^14^. Focusing resources on achieving high coverage for annual treatment may be more resource-effective than biannual MDA with lower coverage ^23^.

Loiasis co-endemicity presents a severe impediment for LF elimination programs, as both diethylcarbamazine and ivermectin can cause severe side effects in people highly infected with loiasis. The World Health Organization-recommended strategy for such areas is twice-yearly MDA with albendazole alone. Early modelling of twice-yearly albendazole, guided by limited empirical data, suggests that the required treatment duration under this strategy will be longer than for annual MDA with IA or DA ^9^. Test (for loiasis)-and-not-treat (those with too high *L. loa* mf) could be an alternative strategy, if LoaScopes or other rapid diagnostics to test for loiasis become available ^33^. As only a small proportion of the population has to be excluded because of high *L. loa* mf density ^34^, this strategy will likely be successful in almost the same timespan as with standard MDA if adherence is equally good. However, this strategy may be relatively costly ^35^.

### Ability to sustain achievement of the goal

An important question is what measures are needed after the cessation of MDA to sustain the achievements. Field studies showed that low-level transmission can continue after passing TAS ^36^. Indeed, TAS can be passed with some residual infection remaining and, moreover, small foci with residual infection may be missed by TAS methodology (see above). Residual infection remaining after MDA cessation can lead to resurgence and reintroduction in areas that had been freed of LF, as was shown by a modelling framework for LF in American Samoa ^17^, although these findings are not necessarily generalizable to other areas with different transmission conditions. Therefore, even after validating elimination of LF as a public health problem by passing the 3 ^rd^ TAS, some form of post-validation surveillance is required for early detection of possible resurgence. Quantitatively-informed guidance is needed for post-validation surveillance and for measuring elimination of transmission. ****

The risk of some residual infection remaining after stopping MDA will vary within treatment areas due to geospatial variation in baseline endemicity, transmission conditions (vector species, biting rate, heterogeneity in the exposure to vectors, etc.), or uptake of interventions. The risk of resurgence depends on the abundance of residual infections and the epidemiological setting. Theoretically, there is threshold prevalence below which the mating probability of any given adult worm is too low to sustain transmission, so that transmission will eventually cease to occur (elimination of transmission) even in the absence of further interventions. This breakpoint depends on specifics of the epidemiological setting, including vector species characteristics, vector abundance or local biting rate, heterogeneity in exposure to mosquito bites within the human population, density dependence in transmission processes, etc. For example, fewer vectors (e.g. through control) increase the threshold ^10, 11, 37^, whereas assortative mixing will decrease the threshold ^38^. Modelling work has been conducted to assess breakpoint thresholds for mf prevalence, antigen prevalence and third-stage larvae (L3) prevalence in the vector population. Mf prevalence threshold values can be far below 1% and vary from site to site ^11, 24, 29^, and are unmeasurable in the current TAS framework. L3 prevalence in mosquitoes could be the most sensitive indicator of transmission ^11^, and sequential sampling approaches based on infection in vectors could be more sensitive ^29^. Xenomonitoring gives a real-time indication of parasite presence and levels in communities ^39– 41^. When prevalence is above the breakpoint, transmission can still die out stochastically. However, the risk of failure increases with increasing prevalence ^42^.

Better understanding of spatial variations in transmission and uptake of interventions is critical for understanding which settings are at greatest risk of resurgence. Strengthening vector control during the endgame could reduce this risk and overcome site-to-site variation in timelines to elimination ^11^.

### Considerations of cost

A recent systematic review found that the WHO recommended strategies for LF elimination are consistently cost-effective or cost-saving across a wide range of settings and assumptions ^43^. Model projections suggest that 175 million disability-adjusted life years (DALYs) were potentially averted by the first 15 years of the GPELF, saving a possible $100.5 billion (USD) over the lifetime of the benefited cohorts ^44^. Models suggest that the increased biannual treatment costs will be compensated for by shorter timescales ^23^. In poor coverage areas, enhancing coverage is the most cost-effective way to accelerate success ^23^.

## What are the main risks that need to be mitigated to achieve and maintain the stated goals?

Countries that have not started MDA will require accelerated scale-up to achieve 2030 goals ^45^. The current TAS-design is likely insufficient to guarantee the eventual elimination of transmission in all the different settings; hence, clear post-MDA and post-validation surveillance guidelines are required. Some experience on this is available from low-endemic areas. Some highly endemic areas are a long way from reaching the epidemiological targets for elimination as a public health problem. Weak post-validation surveillance (e.g. due to lack of guidance, resources or motivation to find cases) may lead to late detection of resurgence and the achievement of <1% mf prevalence may be lost.

## Immediate priorities for future modelling

Priorities for future modelling have been identified in discussions with representatives from WHO. Table 2 lists these priorities and briefly characterizes how modelling can help to address them.

**Table 2.** Priorities questions identified in discussion with WHO and how modelling can help to address them.

Priority question / issue identified in discussion with WHO	How can modelling address this?
1. Identify countries where intensification of interventions is required to reach the target of elimination as a public health problem by 2030.	Use models to make spatially-explicit predictions of the end-year of MDA, considering baseline endemicity (as estimated by a geospatial model) and history of MDA (start year, frequency, achieved coverage). Compare model- based spatially-explicit predictions of the end-year of MDA with the end-year as estimated by WHO using other approaches.
2. What is the probability that there are still locations with mf prevalence >1%, in spite of passing TAS? How frequently does this occur and what are the drivers of this? Identify countries and subnational areas at highest risk.	Apply the models to simulate trends in infection in large number of villages together forming an evaluation unit, assuming that the same MDA was applied in all villages (MDA regimen, duration of MDA) and assuming variation in both baseline endemicity and achieved MDA coverage. Sample settings and children within settings according to TAS methodology. Assess under which circumstances and how frequently there are still locations with >1% mf prevalence, despite passing TAS. Investigate the same under modified TAS sampling schemes (e.g. improving site selection, adjusting critical threshold, using different diagnostics).
3. Are current criteria for stopping MDA and the design of post-MDA surveillance appropriate after treatment with IDA (instead of DA)? If not, how should they be adapted?	Similar to 2), but focusing specifically on sites using IDA instead of DA. Work with data collected in clinical and field trials of IDA to model dynamics of antigen in response to IDA in different settings and the simulate sampling.
4. What are the best ways to design post-validation surveillance to detect resurgence with limited resources?	Simulating dynamics of transmission once the <1% mf population prevalence has been met to estimate probability of and timeline to true elimination of transmission or resurgence. Identify the main drivers and early indicators of elimination of transmission and resurgence.

MDA, mass drug administration; TAS, transmission assessment surveys; IDA, ivermectin, diethylcarbamazine and albendazole; DA, diethylcarbamazine and albendazole.

## Data availability

No data are associated with this article.
