# Supplementary material for: The roadmap towards elimination of lymphatic filariasis by 2030: insights from quantitative and mathematical modelling
Source: Gates Open Res. 2019 Sep 13;3:1538. [Version 1] doi: 10.12688/gatesopenres.13065.1 (PMC6833911; doi:10.12688/gatesopenres.13065.1)
Supplement: Peer review history

# Peer review: Reviewer response for version 1

**DOI:** 10.21956/gatesopenres.14196.r27999

**Published:** 2019-11-05

**Authors:** Ryan E. Wiegand

**Affiliations:** 1 Division of Parasitic Diseases and Malaria, Center for Global Health, Centers for Disease Control and Prevention, Atlanta, GA, USA

Disclaimer:

- The findings and conclusions in this report are those of the reviewer and do not necessarily represent the views of the CDC.

 

Summary:

- This open letter written by the Lymphatic Filariasis Group of the NTD Modelling Consortium contains a review of the mathematical models developed for lymphatic filariasis (LF) and their accomplishments to date. As stated in the background, the modelers in this consortium take the opportunity to reflect on the proposed 2030 goal targets, especially the perceived risks of not achieving global elimination of LF as a public health problem.

 

General comments:

- For a high-level review, sufficient detail is provided by the authors on the models. The article is clear and well-written and quantitative details are largely contained within the "Models for lymphatic filariasis" section. As a prior reviewer noted (Graves, 2019 ^1^), most of the career modelers for LF are included in this group, which can sometimes make it more challenging for incorporating different viewpoints, especially those based on models from outside of the Consortium. That said, the authors do an excellent job of highlighting model findings and how they have been translated into practice.
- The authors presented thoughtful plans for dealing with residual microfoci (or hotspots) of LF infection and their relation to cessation criteria for MDA. There is recent evidence of residual microfoci (or hotspots) of LF infection after many years of treatment (Biritwum *et al.*, 2016 ^2^, Lau *et al.*, 2017 ^3^ and Ahorlu *et al.*, 2018 ^4^). Priorities 2 and 3 of the modeling priorities would provide useful information on hotspot frequency, especially regarding evaluating sampling schemes. Additionally, it may be interesting for the modeling Consortium to test whether survey and response (Harris and Wiegand, 2017 ^5^) or surveillance and response (Bergquist *et al.*, 2015 ^6^) is a viable strategy in post-TAS LF countries for eliminating hotspots and as a possible design for post-validation surveillance (priority question #4).
- I was surprised not to see a priority question on improved coverage. A theme throughout this letter is the need for sufficient chemoprophylaxis coverage and the authors importantly note that intervention modifications are not a substitute for poor coverage. Thus, coverage remains the biggest risk to achieving the 2030 targets. Ideally, mathematical modeling can be utilized to provide more tangible recommendations for programs to increase coverage. The authors note some of the successes; I wonder whether it is possible for modeling to provide insight on the best strategies to increase community participation (King *et al.*, 2011 ^7^ and Deardorff *et al.*, 2018 ^8^) or to assess the feasibility and cost of broad implementation of special surveys that have been shown to increase coverage and compliance (Krentel *et al.*, 2016 ^9^).
- Also, I was unsure about how the spatially-explicit predictions mentioned in priority question #1 would be generated. The authors mention an agent-based model (Xu *et al.*, 2019 ^10^) in the “Models for lymphatic filariasis” section. Maybe the plan is to use the same model to address priority question #1. I encourage the NTD Modelling Consortium to see if geostatistical models can be used here. Model-based geostatistics (Diggle *et al.*, 2002 ^11^) have been implemented for prevalence mapping (Diggle and Giorgi, 2016 ^12^) and spatial statistical models have been used recently for LF (Moraga *et al.*, 2015 ^13^, Eneanya *et al.*, 2018 ^14^ and Giorgi *et al.*, 2018 ^15^). This includes a model integrating mathematical and statistical modeling (Moraga *et al.*, 2015 ^13^). The authors commented that, “better understanding of spatial variations in transmission and uptake of interventions is critical for understanding which settings are at greatest risk of resurgence,” in relation to vector control. Potentially geostatistical models could be used to find covariates associated with risk of resurgence and used to predict the probability resurgence for other locations.

 

Specific comments:

1. The first sentence of the last paragraph of the Background section seems like a run-on sentence.
2. Table 1, line 6, “main” should be “maintain”.
3. Table 2, title, “Priorities” should be “Priority”.

I confirm that I have read this submission and believe that I have an appropriate level of expertise to confirm that it is of an acceptable scientific standard.

## References

[1] : Peer Review Report For: The roadmap towards elimination of lymphatic filariasis by 2030: insights from quantitative and mathematical modelling [version 1; peer review: 1 approved]. *Gates Open Research*.2019;3(1538) : 10.21956/gatesopenres.14196.r27852 10.21956/gatesopenres.14196.r27852 Reference source PMC683391131728440

[2] : Persistent ‘hotspots’ of lymphatic filariasis microfilaraemia despite 14 years of mass drug administration in Ghana. *Transactions of The Royal Society of Tropical Medicine and Hygiene*.2016;110(12) : 10.1093/trstmh/trx007 690-695 10.1093/trstmh/trx007 28938053

[3] : Detecting and confirming residual hotspots of lymphatic filariasis transmission in American Samoa 8 years after stopping mass drug administration. *PLoS Negl Trop Dis*.2017;11(9) : 10.1371/journal.pntd.0005914 e0005914 10.1371/journal.pntd.0005914 28922418PMC5619835

[4] : Community perspectives on persistent transmission of lymphatic filariasis in three hotspot districts in Ghana after 15 rounds of mass drug administration: a qualitative assessment. *BMC Public Health*.2018;18(1) : 10.1186/s12889-018-5157-7 10.1186/s12889-018-5157-7 PMC580985829433461

[5] : Detecting infection hotspots: Modeling the surveillance challenge for elimination of lymphatic filariasis. *PLoS Negl Trop Dis*.2017;11(5) : 10.1371/journal.pntd.0005610 e0005610 10.1371/journal.pntd.0005610 28542274PMC5453617

[6] : Surveillance and response: Tools and approaches for the elimination stage of neglected tropical diseases. *Acta Trop*.2015;141(Pt B) : 10.1016/j.actatropica.2014.09.017 229-34 10.1016/j.actatropica.2014.09.017 25301340

[7] : Improving community participation to eliminate lymphatic filariasis in American Samoa. *Acta Trop*.2011;120 Suppl 1: 10.1016/j.actatropica.2010.08.021 S48-54 10.1016/j.actatropica.2010.08.021 20932818

[8] : Strategies to improve treatment coverage in community-based public health programs: A systematic review of the literature. *PLoS Negl Trop Dis*.12(2) : 10.1371/journal.pntd.0006211 e0006211 10.1371/journal.pntd.0006211 29420534PMC5805161

[9] : Improving Coverage and Compliance in Mass Drug Administration for the Elimination of LF in Two 'Endgame' Districts in Indonesia Using Micronarrative Surveys. *PLoS Negl Trop Dis*.2016;10(11) : 10.1371/journal.pntd.0005027 e0005027 10.1371/journal.pntd.0005027 27812107PMC5094770

[10] : GEOFIL: A spatially-explicit agent-based modelling framework for predicting the long-term transmission dynamics of lymphatic filariasis in American Samoa. *Epidemics*.2019;27: 10.1016/j.epidem.2018.12.003 19-27 10.1016/j.epidem.2018.12.003 30611745

[11] : Model-based geostatistics. *Journal of the Royal Statistical Society: Series C (Applied Statistics)*.2002;47(3) : 10.1111/1467-9876.00113 299-350 10.1111/1467-9876.00113

[12] : Model-Based Geostatistics for Prevalence Mapping in Low-Resource Settings. *Journal of the American Statistical Association*.2016;111(515) : 10.1080/01621459.2015.1123158 1096-1120 10.1080/01621459.2015.1123158

[13] : Modelling the distribution and transmission intensity of lymphatic filariasis in sub-Saharan Africa prior to scaling up interventions: integrated use of geostatistical and mathematical modelling. *Parasit Vectors*.2015;8: 10.1186/s13071-015-1166-x 560 10.1186/s13071-015-1166-x 26496983PMC4620019

[14] : Environmental suitability for lymphatic filariasis in Nigeria. *Parasit Vectors*.2018;11(1) : 10.1186/s13071-018-3097-9 513 10.1186/s13071-018-3097-9 30223860PMC6142334

[15] : Bivariate geostatistical modelling of the relationship betweenLoa loa prevalence and intensity of infection. *Environmetrics*.2018;29(5-6) : 10.1002/env.2447 10.1002/env.2447

# Peer review: Reviewer response for version 1

**DOI:** 10.21956/gatesopenres.14196.r27852

**Published:** 2019-10-02

**Authors:** Patricia M. Graves

**Affiliations:** 1 College of Public Health, Medical and Veterinary Sciences, James Cook University, Cairns, Queensland, Australia

The letter reviews a large body of work (primarily conducted by the authors) on mathematical models of lymphatic filariasis (LF) with aim to reflect on the feasibility of eliminating LF as a public health concern by 2030. The authors present few other views and opinions to date since the authors represent the main modelling groups contributing to the topic. However, an earlier paper by Rebollo and Bockarie 2017 Trends Parastol is not cited, nor do they discuss why the previous prediction of elimination by 2020 was overoptimistic. The letter discusses the key challenges and unknowns that have and will continue to complicate the achievement of this goal. The authors conclude that goal is technically feasible, outlining the major improvements and upscaling of interventions that would be required. They discuss how reducing prevalence below 1% (part of the WHO definition of eliminating LF as a public health concern) relates to local elimination of transmission and the challenges inherent in maintaining prevalence below this level and designing surveillance programs that are sensitive to detect possible resurgence in a timely manner. To the best of our knowledge, all factual statements are correct and nearly all are adequately supported by arguments from the literature. Furthermore, the next steps of modelling of LF are mostly explained clearly. We point out a few of the exceptions to this below.

 Table 1: “Is the new target technically feasible under the current disease strategy? Yes, provided that coverage is high enough, systematic non-adherence is low, and mass drug administration (MDA) has already started.” I agree that the modelling evidence suggests that the new target is achievable given these requirements. However, the requirements seems to contradict what the authors state in the background section i.e. that MDA *has not begun* in some countries. For example, LYMFASIM, TRANSFIL, and EPIFIL predict that in African settings where *Loa loa* is coendemic, even biannual MDA will take on average more than 10 years to reduce Mf prevalence below 1% if initial Mf prevalence exceeds 15%, 25%, or 30% respectively (Stolk et al. 2018 ^2^). Furthermore, there are also several high conflict or challenging countries (e.g. PNG) that are unlikely to start MDA in all endemic IUs until well after 2020.

 “Clearly, GPELF’s 2020 targets will not be met everywhere.” I agree with this statement. However, while the letter to this point clearly states that we are not there now, the letter to this point doesn’t argue why success can’t be pulled off at short notice. A line stating that areas that haven’t begun MDA will need X or more years would correct this.

 “Uncertainty about the dynamics of, and association between, different infection indicators makes it difficult to quantify the risk of resurgence associated with signals of residual transmission.” I believe it would be more helpful to be more explicit by briefly elaborating on the ‘different infection indicators’ – e.g. Antigen vs Mf prevalence.

 “L3 prevalence in mosquitoes could be the most sensitive indicator of transmission” The following reference calculates critical prevalence thresholds (breakpoint values) below which transmission will die out without further interventions. The breakpoint for prevalence of L3 larvae mosquitos are consistently lower than prevalence of Mf in humans. I believe this is what the authors mean by ‘sensitive indicator’. The context of the sentence is a discussion of breakpoint prevalence values, however the connection to breakpoints was not immediately clear to me on a first read through. Perhaps it is better to put this in terms consistent with the rest of the paragraph, i.e. something like “L3 prevalence in mosquitoes could be the most sensitive indicator of transmission that will lead to resurgence if unchecked, as the breakpoint for L3 prevalence is much lower than the breakpoint for Mf in humans.”

 “However, using IDA is not a solution for poor coverage.” Though I think I agree with what the authors mean to communicate, it should be noted that if IDA is better than DA at all IDA surely can certainly go *some* way to makeup for poorer coverage. The authors should elaborate and cite examples or models that illustrate this.

 “Thirdly, models predicted that the required treatment duration can also be reduced considerably by treating biannually (i.e. twice per year) instead of annually if coverage remains the same, assuming that a) the second round reaches some people who were missed in the first round and b) people treated twice benefit from additional chemotherapeutic effects on worms and mf” I do not understand the reason for stating these two assumptions here – aren’t these also assumptions of the other studies/models with more than one round of MDA?

 Table 2 Row 1. I agree that spatially-explicit models can provide very useful insights, but given the flight range of mosquitos and typical human movements, wouldn’t these kinds of model be most helpful to identify *regions within* *countries* (rather than whole countries) where intensification of interventions is required to reach the target of elimination?

 Finally, we have also noted a few minor language issues

 Table 3 column two. I do not understand the last part of the sentence “and the simulate sampling”

 “WHO is currently developing new targets and milestones beyond 2020, which should be aligned with the sustainable development goals (SDGs) and should be ambitious, evidence-based and realistic” Not clear whether the authors are asserting that these targets and milestones *should* be such and such, or whether the this is one of the intentions of WHO.

 Table 1:”Are additional interventions (e.g. vector control) required to main the achievements?” main -> maintain

I confirm that I have read this submission and believe that I have an appropriate level of expertise to confirm that it is of an acceptable scientific standard.

## References

[1] : Can Lymphatic Filariasis Be Eliminated by 2020?. *Trends Parasitol*.33(2) : 10.1016/j.pt.2016.09.009 83-92 10.1016/j.pt.2016.09.009 27765440

[2] : Are Alternative Strategies Required to Accelerate the Global Elimination of Lymphatic Filariasis? Insights From Mathematical Models. *Clinical Infectious Diseases*.2018;66(suppl_4) : 10.1093/cid/ciy003 S260-S266 10.1093/cid/ciy003 PMC598279529860286
